# Health related quality of life among adolescents with sickle cell disease in Saudi Arabia

**DOI:** 10.4314/pamj.v8i1.71057

**Published:** 2011-02-15

**Authors:** Mostafa Abdel-Monhem Amr, Amin Tarek Tawfik, Ahmed Omar Al-Omair

**Affiliations:** 1 Department of Clinical Neuroscience, College of Medicine, King Faisal University-Al Hassa, Kingdom of Saudi Arabia; 2 Community Medicine Department, Faculty of Medicine, Cairo University, Cairo, Egypt; 3 Department of Internal Medicine, King Fahd Hospital, Al Hufof, Kingdom of Saudi Arabia

**Keywords:** Sickle cell disease, adolescents, HRQoL, auality of life, disease complications, socio-demographics

## Abstract

**Introduction:**

Increased life expectancy due to recent medical advances has increased the need to understand more fully the quality of life (QoL) in patients with sickle cell disease (SCD) and factors predicting disease adaptation .The objectives of this study were to assess the impairment of health related quality of life (HRQoL) domains in a sample of Saudi Arabian adolescents with SCD.

**Methods:**

A non-probability sample composed of Saudi adolescents with SCD (n=180) aged 14-18 years and comparable age and gender matched healthy controls (n=202). Socio-demographics and disease related data were obtained through personal interview with parents/legal guardians and reviewing patients′ medical records. Selfadministered ‘Short Form-36’ questionnaire was used to assess HRQoL of the included groups.

**Resultats:**

HRQoL showed significant deterioration in adolescents with SCD especially in role physical, general health, and bodily pain domains irrespective of the gender, while female adolescents with SCD demonstrated significant deterioration in emotional wellbeing. Those with SCD-complications showed worse scores along the physical, general health, and emotional wellbeing domains. HRQol scores were negatively associated with increasing age, female gender, rural residence, low family income, presence of disease-related complications and frequent hospital admissions as revealed by multivariate regression analysis.

**Conclusion:**

Saudi adolescents with SCD experience deterioration along all domains of HRQoL especially the physical. Disease related complications and sociodemographic correlates are significant determinants to worse HRQoL among the included adolescents with SCD.

## Introduction

Sickle cell disease (SCD) is a group of inherited red blood cell disorders characterized by the presence of abnormal hemoglobin. The clinical manifestations are diverse and may include vaso-occlusive, hematological and infectious crises [1,2]. SCD is present throughout Saudi Arabia; particularly common in the eastern and southern provinces: Qatif (eastern region) 17.0 %, Gizan (southern region), 10.3%, Ula (Northern region) 8.1 % and Mecca (western region) 2.5 % [3]. Increased life expectancy due to recent medical advances has increased the need to understand more fully the quality of life (QoL) in patients with SCD and factors predicting disease adaptation [4,5]. QoL measures are used not only to assess the psychosocial impact of the disease but also in evaluating the efficacy of medical care [6-9] and it is the goal of health care providers to enhance treatment outcome and restore comfort and well-being of their patients [10]. In particular, health related quality of life (HRQoL) assessments in adolescents with chronic disease condition facilitate doctor–patient communication, they point to areas where patients may experience serious problems, they can be used as diagnostic tools for problem-oriented follow–up care, and the data are strong predictors of survival [11].

Previous research indicated that patients with SCD experienced a lower HRQoL compared to the general adult’s population [12] Dampier et al [13] found that children with SCD as well as their parents scored significantly lower on several HRQoL domains including; general physical, motor and independent daily functioning. Trzepacz et al [14] identified deteriorations in social and school competence for children with SCD, compared to healthy peers, but they did not find an association with disease severity as measured by sickle cell genotype. A number of disease-related factors have been found to affect HRQoL in pediatric SCD. Several studies that examined the influence of various determinants including the role of sociodemographics, disease severity and the presence of complications on HRQoL in SCD patients were carried out in the developed countries [12,13,15,16] while in the developing countries similar studies do not exist. Furthermore, adolescent health is a relatively unexplored component of public health in many developing countries [17,18].

Researches on chronic diseases have indicated that HRQoL varies according to socio-demographic characteristics such as income level, educational status, ethnicity, occupational status, age, and gender, with the disadvantaged groups typically reporting lower HRQoL. This association has been reported for many chronic conditions including cancer [19], HIV infection [20], renal disease [21], and sickle cell disease [15]. Also, studies involving healthy populations have indicated that there is an inverse association between children’s HRQoL and family variables such as low parental education, socio-economic status (SES) [22-24] with low family income contributing to caregiver distress in families of children with chronic conditions [25]. Also, Simon et al [26] reported that HRQoL is poorest for children and youth in lower socioeconomic status groups, those with access barriers, adolescents compared with children, and individuals with medical conditions.

Studies on the relationship of socio-economic variables and the HRQoL in patients with SCD revealed conflicting results. Van den Tweel et al [27] indicated that children of parents with low educational level perceived a significantly better HRQoL. This phenomenon is difficult to explain, since previous research mainly pointed out that high quality of life scores were related to high parental education, or that education had no effect at all [11,28]. Also, previous research has shown that children with low socioeconomic status (SES) functioned worse than children from middle SES backgrounds [29].

Limitations in HRQoL have been documented consistently for youth with SCD, [15,30,31] particularly as children move into adolescence and young adulthood [32,33]. Sickle cell pain, a common manifestation that is recurrent, acute, and unpredictable, may be the most important disease complication associated with deterioration in physical and psychosocial domains of HRQoL [15,34,35]. Palermo et al [32] reported that sickle cell complications (including pain), in addition to child age and gender, are central to physical but not psychosocial HRQoL in their sample of adolescents with SCD. Yet, Panepinto and colleagues [36] found that only pain, no other SCD complications, was associated with the deterioration in the physical domain of HRQoL but not the psychosocial.

Although pain and other sickle cell complications show an association with decrements in engagement in physical activities and in physical domains of HRQoL, documentation of a significant association of pain with psychosocial domains of HRQoL are not consistent [37]. Recently, Brandow et al.

[38] examined HRQoL in children with SCD specifically in relation to painful events at presentation to the emergency room and seven days postdischarge. They found that painful events diminished all domains of HRQoL and that these domains improve after the pain resolves. If these variables (socio-demographic and disease related complications) are crucial in determining HRQoL among adolescent patients with SCD, by controlling of these factors, we may better support them with successful transition to adulthood and with less burden on healthcare services [39]. We specifically hypothesized that adolescents with SCD would have decreased mean scores along the different subscales of the HRQoL measure compared to adolescents without SCD. We also predicted that certain demographic factors (increasing age, gender, low socio-economic status) would be related to HRQoL with males and adolescents from lower SES backgrounds reporting lower quality of life. Additionally, we predicted that adolescents with SCD who experienced disease-related complications, frequent pain episodes, and greater health care utilization would report lower quality of life than adolescents with SCD who did not report these factors. The objective of this study were to assess the impairment of the different domains of HRQoL among Saudi adolescents with SCD compared to healthy peers and to define the relationship between socio-demographic variables, the presence of diseases related complications with the degree of impairment in HRQoL.

## Methods

**Settings**

This study was carried out in Hematology clinics of two general hospitals; King Fahd Hospital in Hofuf and Al Qatif General Hospital in Al Qatif city, Eastern Province, Saudi Arabia. Both hospitals provide secondary level of care and serve about one and half million populations. Adolescents with SCD and their counterparts were selected from these hospitals.

**Patients**

Adolescents with SCD were recruited out of those attending Hematology clinics at both hospitals attending for routine assessment, follow-up and management. Inclusion criteria for adolescents with SCD included: Both genders with age ranged from 14 to 18 years of indigenous Saudi nationality and free form acute or chronic illnesses, handicapping conditions, and cognitive problems (Data were affirmed through the hematology consultants at the clinics and review of medical records). A total of 285 adolescents with SCD were eligible from both hospitals (King Fahd n=132) and Al Qatif (n=153) of which 188 were males and 97 females.

Personal contact and phone calls were used to approach patients’ parents/legal guardians to recruit the eligible SCD adolescents. Out of those approached; parents/guardians of 136 males and 44 females agreed to participate. There was no significant difference in relation to the socioeconomic and disease status of those refused to participate compared to the rest of the sample as revealed from reviewing of available hospital records. Higher refusal was recorded among parents of females with SCD, as the response rate was 45.4% compared to males (72.3%).

Adolescents without SCD: Following the selection of SCD patients, we employed a non-probability sampling method to recruit healthy adolescents for the sake of comparison. Attendees from the two hospitals within the same age range, nationality, and gender and accompanying their relatives at various outpatients clinics.

Exclusion criteria included: Having any chronic physical illnesses or cognitive problems, diagnosed with any hemoglobinopathies or having a positive family history of hemolytic blood diseases and with positive history of acute, chronic illnesses or health related problems within the last four weeks of data collection.

Out of 361 (253 males and 108 females) subjects approached, parents’ (guardians) of 202 adolescents (152 males and 50 females) agreed to participate. The existence of a significant difference in relation to the socio-demographic (other than age and gender) of this group was not confirmed due to lack of information.

Permissions were obtained from hospitals’ authorities as well as our institution after approval of the proposal and data collection tools. Prior orientation of parents/legal guardians was carried out before obtaining of an informed consent form with emphasis on the right of the subject of non-participation. Assents forms were obtained from adolescents after receiving proper orientation. Data confidentiality was maintained all through the study.

**Measurements**

Socio-demographic data were collected through a personal interview with parents/legal guardians of adolescents with SCD and the comparison group using a structured questionnaire to elicit information regarding socio-demographic characteristics including age in years, residence, educational status of parents and subjects and monthly family income in Saudi Riyals. The interviews were carried out on the same day of attending the clinics in the afternoon in a separate room away from the busy clinics by trained interviewers (medical graduates) under the supervision of the investigators.

Disease related data SCD were obtained through available medical records and confirmed by the attending hematology consultants, including: disease genotype, percentage of fetal hemoglobin, SCD related complications and their nature, frequency of painful episodes in the last month, and frequency of emergency hospital admissions in the last year.

Health Related Quality of Life: Following parents/guardians interview, HRQoL was assessed through self-reporting of the Short-Form (SF-36) ‘version 2.0’ [40]. All participants were asked to fill the questionnaire; assistance was provided when necessary for those with reading and/or writing difficulties.

The SF-36 is one of the most common, generic measures of HRQoL and has been employed in numerous studies involving chronic illnesses. The SF-36 questionnaire was designed in the 80s to provide a generic measure of HRQoL of those aged 14 years and older [41]. The translated Arabic version of the SF-36 [42] which has shown reliability with Arabic-speaking population was employed to evaluate HRQoL.

SF-36 is made up of eight subscales, each evaluating a different domain of HRQoL; physical functioning, physical role functioning, bodily pain, general health, vitality and energy, social functioning, role emotional and mental (emotional) health. Subscale scores are calculated from 35 out of the 36 items (one item about self-reported health transition is not included in the scores). SF-36 yielding score values from 0 to 100, higher scores indicate better quality of life. Alpha coefficients were calculated to estimate the internal consistency reliability of each SF-36 subscale. Alpha coefficients were high in the domains of physical functioning, role physical and role emotional among both groups of adolescents. Bodily pain, vitality, emotional well-being and social functioning showed a coefficient of less than .8 while, general health showed the least reliability coefficient among adolescents with SCD.

**Data analysis**

With inclusion of 131 subjects in each group we had more than 90% [43] power to detect 8.0 [12,16], standard deviations difference with precision of ±3.0 along the eight subscales of HRQoL between adolescents with SCD and their comparable group of adolescents without SCD at an alpha level of 0.05 (two sided). Considering a potential refusal rate of 30%, the total sample size would be 170 subjects in each group.

Data were processed and analyzed using SPSS version 16.0 (SPSS Inc. Chicago IL). Analysis was carried out along the SF-36 scoring protocol. Results obtained from HRQoL domains were expressed in mean and standard deviation. Both descriptive and inferential statistics were applied as appropriate.

Statistical tests of significance including t-test for continuous variables, Chi-square and Fisher Exact were used for categorical data to detect difference between groups. Effect sizes in the form of Cohen’s d [44] were calculated as the difference between the means, divided by standard deviation of either group when the variances of the two groups are homogeneous, in our case we employed the pooled standard deviation to calculate the effect size (equals to the root mean square of the two standard deviations for adolescents with and without SCD).

The interpretation based on the average percentile standing of the average of adolescents without SCD relative to the average of adolescents with SCD. Multivariate linear regression models were generated to define the association between socio-demographics and SCD related variables (independent) to the mean scores along HRQoL domains (dependent), standardized β coefficients and P value were reported for each model. P value of < 0.05 was applied as a level of significance.

## Results

**Sociodemographics**

Adolescents with SCD (n=180, 136 males and 44 females) and a comparable group of adolescents without SCD (n=202, 152 males and 50 females) their age ranged from 14 to 18 years with a mean of 16.9± 2.5 years, the mean age of adolescents with SCD was 16.8±3.6 years and the age of their counterpart was 16.9±1.7 years.

Adolescents with SCD have a lower socio-demographic profile in terms of parental educational status and family income compared to adolescents without SCD but without statistical difference (table 1 ).

Adolescents with SCD showed a significant educational delay in terms of excessive failing and school retention while adolescents without SCD was significantly better; 15.0% of adolescents with SCD demonstrated delay (excessively retained in relation to their comparable peers) in the primary (elementary=up to grade 6) education compared to only 2.0% among adolescents without SCD, and 71/81 (87.7%) of adolescents with SCD in the preparatory stage (intermediate= up to grade 9) were delayed compared to 8/39 (21.1%) among adolescents without SCD. This delay was attributed by the parents due to excessive absenteeism from schools in response to frequent hospitalization, emergency admissions, and appointments for checkups.

**Genotypes and disease-related complications**

table 1 displays the encountered SCD genotypes and SCD related complications among the included adolescent with SCD. Painful episodes in the last month were recorded in 56.7 % of cases, and the need for emergency hospital admission during the last year was found in 25.5% of cases. All SCD patients were on Hydroxyurea (HU) therapy, recorded fetal hemoglobin level (in 167/180 cases) had a mean of 13.1±6.4 % (median of 12.5%) with an interquartile range of 19.0%.

**Health Related Quality Of Life**

Domains of HRQoL were significantly deteriorated in adolescents with SCD (table 2 ). The mean scores of adolescents without SCD were above the 93^rd^ percentiles of adolescents with SCD in role physical and general health domains, above the 97^th^ percentiles in the domain of bodily pain, higher than 79^th^ percentiles of adolescents with SCD in physical functioning, and 76^th^ percentile in the social functioning domain.

Male adolescents with SCD scored significantly worse than males without SCD in all domains of HRQoL (table 3). Male adolescents without SCD demonstrated mean scores above the 96^th^ percentiles of adolescents with SCD in role physical and bodily pain domains, above the 93rd percentiles in general health, above the 79^th^ percentiles of physical functioning domains. Role emotional and emotional well-being domains showed less effect sizes, the mean scores among adolescents without SCD were above the 58^th^ and 66^th^ percentiles of the mean of adolescents with SCD respectively.

Adolescent females with SCD achieved lower scores than females without SCD in role emotional, vitality, emotional well-being and social functioning domains but without statistical significance. Adolescent females without SCD had mean scores in the physical functioning and bodily pain above the 95^th^ percentiles of females with SCD, general health scores was above the 90^th^ percentiles and role physical mean scores was above 76^th^ percentiles of adolescents with SCD.

Adolescent females with SCD scored higher than females without SCD in role emotional and vitality, adolescent females with SCD achieved mean scores above the 58^th^ percentiles of females without SCD in both domains (table 3).

Male adolescents with SCD achieved higher scores in the domains of physical functioning, bodily pains and social functioning compared to female patients but without statistical difference. Emotional well-being scores were significantly higher among male adolescents with SCD compared to female patients (table 3).

The mean scores of male adolescents with SCD in emotional well-being domain were above the 66^th^ percentiles for females with SCD. Female adolescents with SCD had higher scores in role physical and role emotional domains compared to males with SCD but without significant difference.

Adolescents without SCD complications had mean scores above the 69^th^ percentiles of those with complications in the domains of role physical and role emotional, above the 62nd percentiles in physical functioning, general health, and emotional well-being domains of the average scores for those with SCD complications (table 4).

Multivariate linear regression models showed that all domains of HRQoL were negatively associated with increasing of age of the adolescent with SCD especially in domains of energy, emotional well-being, social function and general health (table 5).

Female gender was negatively associated with scores in the domain of emotional wellbeing. Urban residence was positively associated with higher vitality score and higher family income was associated with increase in scores of physical functioning domain. Frequent hospital emergency admissions were associated with lower scores in almost all subscales especially physical, role physical and emotional wellbeing domains.

## Discussion

To the authors’ knowledge, this is the first study to assess HRQoL in adolescent patients with SCD in an Arabic community. The data reported here showed that adolescents with SCD experiencing impairment in HRQoL when compared with their healthy peers. The smallest differences between adolescent controls and SCD group were evident in the role emotional and emotional wellbeing domains. Similar to our findings, McClish et al [12] reported that young adult with SCD experienced a poor HRQoL except for mental health. Patel and Palham [45] reported that Indian children with SCD experienced a poor HRQoL along physical, psychosocial and cognitive domains.

It has been suggested that people with chronic diseases may report good psychological well-being as a result of increased social support, religiousness, lack of other stressors, or due to the presence of a “response shift" associated with coping and managing of their illness [46].

In Saudi culture, several factors may explain the process of psychological coping among adolescents with SCD. First, the extended family pattern in Saudi Arabia; family provide people suffering from anxiety the presence of “safe persons” around them and hide active symptoms of anxiety, maintaining them functional in a compensatory behavior [47]. Adolescence is typically a period of rapid physical, cognitive and psychosocial change that takes place in the context of shifting relationships and roles within the family [48]. The Family Adjustment and Adaptation Response (FAAR) model highlights how adaptation to chronic childhood illness is explained in the balance of family demands (stressors from individual, family, and community sources) as well as family resources such as positive parent-child relationships, family functioning, and active coping and positive beliefs/attitudes [49]. Second, the health care management policy in Saudi Arabia, where the services are free of charge and all SCD patients received Hydroxyurea (HU) which can improve some domains of HRQoL including social function, general health perception and pain recall [34]. Finally, recorded fetal hemoglobin level in this study had a mean of 13.1±6.4 % (median of 12.5%) with an interquartile range of 19.0%, higher than that reported in patients with African haplotypes [50]. Pearson [51] and Perrine et al. [50] found that serious complications (i.e. jaundice, splenectomy, and hematuria) occurred in only 6% to 25% of Saudi patients compared to North American Blacks.

Despite the increased attention paid to determine factors that affect QoL in SCD [13,45,52] fewer studies addressed the influence of gender. In our study, female participants was less and we speculated that this may be due to the disease associated stigma and fear of disclosure as many females with SCD preferred to receive disease management in private health facilities where more confidentiality is provided.

Despite increased attention being paid to determine factors that affect the quality of life in SCD, there are few studies that address the influence of gender. Some studies suggested that females have a poorer quality of life than their male counterparts [29] while, others did not reveal gender difference [30]. There is also suggestion that gender may influence the way adolescents’ perceiving of health [31].

Among our adolescents with SCD, gender differences in HRQoL existed between males and females. Adolescent girls with SCD achieved significantly lower scores in physical functioning, role physical, bodily pain and general health domains. Ironically, they have higher score along social function and emotional well-being compared to their healthy controls.

Studies dealt with behavior adjustment in children with chronic diseases showed conflicting results in relation to the effect of gender. Zandieh et al

[53] found that QoL in Iranian boys with bronchial asthma were more deteriorated than females.

While in Swedish study, Hanberger et al. [54] reported lower Generic HRQoL and Diabetes-specific HRQoL mean scores in adolescent girls with type 1 diabetes mellitus than boys. HRQoL did not show a significant relationship with SCD genotype. The relationship between SCD genotype, disease severity and HRQoL may have been mediated by other variables, particularly disease-related complications. In addition, some of HRQoL subscales were significantly deteriorated with increasing hospitalization frequency. Anie et al [52] found a relationship between pain and some HRQoL domains ‘physical, social functions, mental and general health’ while others [12] found no significant association with health care utilization measures.

In this study nearly all domains of HRQoL were negatively associated with increasing age of adolescent with SCD especially energy, emotional wellbeing, social functioning and general health domain. Baskin et al. [31] found that there was an interactive effect between age and disease adjustment which become more problematic as SCD patient mature with additional difficulties as the disease worsens in severity (e.g., increase in vaso-oclusive episodes). Furthermore, physical manifestations of SCD notably in the form of abnormal sexual and physical development, as evidenced by delayed growth and puberty, priapism and short stature may add more difficulties to the growing patient.

Adolescents with SCD in our study reported significant educational delay due to frequent hospitalization, emergency admissions, and appointments for checkup. This is consistent with other studies reported that adolescents with SCD commonly experience role limitation due to emotional problems with subsequent educational problems [45,55].

Rural residence was a significant negative predictor for vitality and pain of HRQoL subscales. There are limited studies on rural/ urban differences in QoL among patients with SCD. Telfair et al [55] have shown that rural patients had lower health service access and utilization with lower physical functioning. Future researches should investigate the role the differential levels of social support, coping styles, and perceptions of illness in urban-rural differences in QoL among SCD patients.

Another risk factor for lower HRQoL among our patients was the low family income. Saudi youth with lower socioeconomic standard were more likely to have lowered physical functioning scores. Children living under poorer economic conditions are more prone to anxiety, depression and negative thinking with potential exaggeration with adolescents with SCD through increasing exposure to stressful experiences and decreasing psychological and social coping resources [56]. Panepinto et al [57] have found a significant negative effect of poverty and disease severity on the HRQoL among pediatric patients and it was chiefly affecting the physical functions of the included children with SCD.

Health care providers have limited ability to improve the poverty status of the affected patients but improvement of disease status and prevention of disease complications may help in improvement of their well-being especially physical wellbeing [2].

The combined effects of SCD-related pain and low socioeconomic status [56] on school attendance may limit opportunities for physical education with subsequent reduction of physical HRQoL.

Socio-demographic variables were found to adversely affect the HRQoL in patients with chronic conditions as reveled by several cross-sectional studies, yet prospective studies are required to better elucidate the predictive value of family variables. Barakat et al [57] found that family resources were not significant predictors for QoL among patients with childhood cancer. Furthermore, they stated that the potential negative impact of lower family education, income, employment, and function may be indirect and dynamic over time.

**Study limitations**

The contributions of this investigation must be considered within the following limitations: Low participation of female adolescents with SCD could be explained while considering the socio-cultural and the disease associated stigma that may force females to refrain from participation due to fear from disclosure. In addition most adolescent females with SCD seeking care at other facilities ‘private sector’ where confidentiality is maintained. We drew our population especially the control group, from a non-probability sample, which may have biased our results with subsequent impact on their generalizability.

## Conclusions

Adolescents with SCD experience health related quality of life worse along several domains of HRQOL including physical functioning, role physical, bodily pain and general health compared to their comparison group of apparently healthy adolescents. Socio-demographics including older age of the adolescents with SCD, female gender, rural residence, low family income and the occurrence of disease-related complications were associated mainly with deterioration of physical rather than mental health of our sample of adolescents with SCD.

Controlling of possible modifiable determinants, e.g., disease-related complications and some socio-demographics that affect the HRQoL among adolescents with SCD might improve these patients’ outcome with smooth transition to adulthood. HRQoL assessments in adolescents with SCD facilitate doctor–patient communication, point to areas where patients may experience serious problems during this transitory phase, can be used as diagnostic tools for follow–up care, and assessing the efficiency of different treatment modalities.

## Competing interests

The authors declared they have no competing interests. Also there are no sources of funding.

## Authors’ contribution

MA: Study concept, design of the questionnaires, data discussion manuscript writing, and editing. TTA: Study concept, Statistical analysis, data collection, manuscript writing and review. OAA: Data collection and: helped in manuscript review and editing manuscript writing and review.

## Acknowledgments

The authors would like to thank Dr Abdullah K Al Arfaj and Dr Mohammed S Ibrahim, for their sincere and valuable collaboration and assistance during conduction of this research.

## Figures and Tables

**Table 1:** Sample characteristics of the included adolescents with sickle cell disease (SCD) and their comparable healthy adolescents

	**Adolescents with**	**Adolescents**	**Total (N=382)**	
**Characteristics**	**SCD (N=180)**	**without**	**SCD No (%)**	**P value^a^**
	**No (%)**	**(N=202)**		
		**No (%)**		
**Age groups (years)**				
14-15	68(37.8)	53(26.2)	121(31.7)	0.183
16-17	71(39.4)	81(40.1)	152(39.8)	
18	57(31.7)	68(33.7)	125(32.7)	
**Residence**				
Urban	132(73.3)	158(78.2)	290(75.9)	0.265
Rural	48(26.7)	44(21.8)	92(24.1)	
**Educational status**				
Primary	27(15.0)	4(2.0)	31(8.1)	0.008†
preparatory	81(45.0)	39(19.3)	120(31.4)	
Secondary	72(40.0)	159(78.7)	231(60.5)	
**Father educational status**				
< Secondary	101(56.1)	94(46.5)	195(51.0)	0.0480
Secondary or higher	78(43.9)	108(53.5)	186(49.0)	
**Mother educational status**				
< Secondary	106(58.9)	109(54.0)	215(56.3)	0.332
Secondary or higher	74(41.1)	93(46.0)	167(43.7)	
**Family income in Riyals**				
< 2500	26(14.4)	28(13.9)	54(14.1)	0.395
2500- < 6000	97(53.9)	96(47.5)	193(50.5)	
≥ 6000	57(31.7)	78(38.6)	135(35.3)	
**Genotypes**				
Hb SS	146(81.1)	-	-	
Sickle-β+ Thalasemia	26(14.4)	-	-	
Sickle-β0 Thalasemia	5(2.8)	-	-	
Hb SC	3(1.7)	-	-	
**Disease-related complications**				
Vaso-occulsive	29(43.3)	-	-	
Infections	24(35.8)	-	-	
Acute chest syndrome	3(4.4)	-	-	
Brain infarction	6(9.0)	-	-	
				
Cholethiasis	5(7.5)	-	-	
More than one complication	27(40.3)	-	-	
**Painful crisis in the last month**	46(25.5)	-	-	
**Emergency admission last year**	46(25.5)	-	-	

^a^Chi Square

**Table 2:** Comparison of Health related quality of life “SF36 subscales” between adolescents with sickle cell disease (SCD) and the comparison group

	**Adolescents with SCD (N=180)**		**Adolescents without SCD (N=202)**			
**SF-36 subscales**	**Mean ± SD**	**Alpha^a^**	**Mean ± SD**	**Alpha**	**Effect size Cohen’s *d***	P value
**Physical functioning**	58.34±20.76	0.849	80.25±23.45	0.901	0.988	0.001
**Role physical**	23.10±33.70	0.852	76.36±33.85	0.801	-1.570	0.001
**Role emotional**	43.04±41.43	0.870	51.32±41.27	0.803	-0.200	0.051
**Bodily pain**	33.74±27.23	0.731	81.26±20.30	0.783	-1.978	0.001
**Vitality**	46.51±18.03	0.714	54.73±17.60	0.812	-0.461	0.001
General health	37.58±22.04	0.694	66.81±14.65	0.781	-1.562	0.001
**Emotional well-being**	53.89±18.32	0.740	61.19±18.32	0.801	-0.398	0.001
**Social functioning**	44.73±24.75	0.710	62.31±20.77	0.721	-0.769	0.001

SD=Standard Deviation, ^a^Chronbach’s alpha correlation coefficient

**Table 3:** Health related quality of life domains (mean ±SD) among the included adolescents in relation to presence of sickle cell disease (SCD) and gender

**SF-36 subscales**		**Males (N=288)**				**Females (N=94)**		
	**Adolescents with SCD (N=136)**	**Adolescents without SCD (N=152)**	**Effect size Cohen’s d**	**P value**	**Adolescents with SCD (N=44)**	**adolescents without SCD (N=50)**	**Effect size Cohen’s d**	**P value**
**Physical functioning**	59.96±21.23	79.48±24.27	-0.856	0.001	53.41±18.58	84.67±17.95	-1.711	0.001
**Role physical**	20.65±32.94	79.10±31.84	-1.804	0.001	30.68±35.30	60.83±40.83	-0.789	0.001
**Role emotional**	43.72±42.77	55.23±40.69	-0.275	0.061	48.82±12.93	42.89±27.90	0.273	0.199
**Bodily pain**	35.33±28.66	82.97±19.00	-1.959	0.001	28.75±21.72	71.50±24.68	-1.838	0.001
**Vitality**	46.59±18.86	57.12±16.12	-0.600	0.001	46.25±15.33	41.00±19.63	0.289	0.201
**General health**	39.02±22.96	68.72±13.67	-1.571	0.001	33.07±18.37	55.83±15.49	-1.339	0.001
**Emotional well-being**	55.51±18.62	63.33±17.35	-0.435	0.001	48.81±12.93	48.93±19.19	-0.007	0.975
**Social functioning**	45.10±25.75	64.84±18.13	-0.435	0.001	43.69±21.55	47.83±23.83	-0.182	0.449

SD=Standard Deviation

**Table 4:** Health related quality of life scores (mean ±SD) in relation to presence of diseaserelated complications among adolescents with sickle cell disease

	**Sickle cell disease-related complications^a^**		**Effect size**	
**SF 36 subscales**	**Yes (N=67)**	**No (N=113)**	**Cohen’s *d***	**P value**
**Physical functioning**	56.06±21.21	62.69±19.51	-0.325	0.041
**Role physical**	16.95±31.33	34.92±35.22	-0.539	0.001
**Role emotional**	35.88±41.61	56.61±38.16	-0.519	0.001
**Bodily pain**	32.41±24.44	36.55±31.94	-0.145	0.333
**Vitality**	44.87±17.64	49.60±18.63	-0.261	0.094
**General health**	35.04±21.54	42.46±22.47	-0.337	0.031
**Emotional well-being**	51.69±17.42	58.16±17.43	-0.371	0.019
**Social functioning**	43.39±24.84	47.62±24.53	-0.171	0.275

SD=Standard Deviation, ^a^Includes; vaso-occlusive crises, infections, acute chest syndrome, brain infarction, cholethiasis and others

**Table 5:** Linear regression analysis models between mean scores of SF36 domains and sociodemographic and diseaserelated characteristics in adolescents with sickle cell disease (N=180)

**Independent**	**SF-36 domains, Standardized β coefficients and (P value)**							
**variables**	**Physical functioning**	**Role physical**	**Role emotional**	**Energy and vitality**	**Emotional**	**Pain**	**Social functioning**	**General**
**Age**	-.136(0.771)	-.146(0.157)	-.136(0.190)	-.175(0.026)	-.162(0.038)	-.198(0.011)	-.303(0.001)	-.250(0.016)
**Gender**	.118(0.105)	-.141(0.144)	-.058(0.550)	.021(0.780)	-.168(0.024)	-.112(0.127)	.051(0.482)	.092(0.340)
**Residence**	.088(0.224)	.130(0.137)	-.029(0.763)	.151(0.042)	.043(0.556)	.216(0.003)	-.077(0.107)	.020(0.638)
**Educational status**	.047(0.539)	.050(0.748)	.192(0.030)	.112(0.145)	.043(0.655)	.041(0.674)	.023(0.685)	.047(0.683)
**Family income**	.147(0.041)	.137(0.121)	.081(0.831)	.051(0.873)	.126(0.091)	.025(0.831)	.093(0.481)	.143(0.098)
**Hospital admissions**	-.159(0.012)	-.150(0.040)	-.023(0.631)	-.087(0.739)	-.182(0.033)	.152(0.033)	-.126(0.461)	-.167(0.041)
**Genotype**	-.121(0.214)	-.121(0.206)	-.095(0.323)	-.075(0.444)	-.024(0.801)	.052(0.577)	-.141(0.361)	-.090(0.672)
**Complications**	-.162(0.011)	-.234(0.008)	-.271(0.004)	-.100(0.177)	-.141(0.052)	-.101(0.132)	-.095(0.300)	-.182(0.043)
**R^2^**	.201	.235	.132	0.099	.129	.171	.190	.137
**F test**	6.441	5.680	4.407	2.760	2.547	3.543	4.039	2.732
**Constant**	67.7	58.168	97.76	63.32	85.65	77.38	110.98	90.77

Age and frequency of hospital admissions were entered as continuous variables. Gender (0=female, 1=male), residence (0=rural, 1=urban), educational status (0=primary, 1=intermediate, 2=secondary).Family income (0=  <  6000, 1= 6000 or more Saudi Riyals), genotype (0=SS, 1=others), complications (0=no 1=yes).
